# Supplementary material for: Optimal Woven EndoBridge (WEB) Device Size Selection Using Automated Volumetric Software
Source: Brain Sci. 2021 Jul 8;11(7):901. doi: 10.3390/brainsci11070901 (PMC8307121; doi:10.3390/brainsci11070901)
Supplement: Supplementary file 1 [file brainsci-11-00901-s001.zip › brainsci-1278795-supplementary.pdf]

Supplemental Material

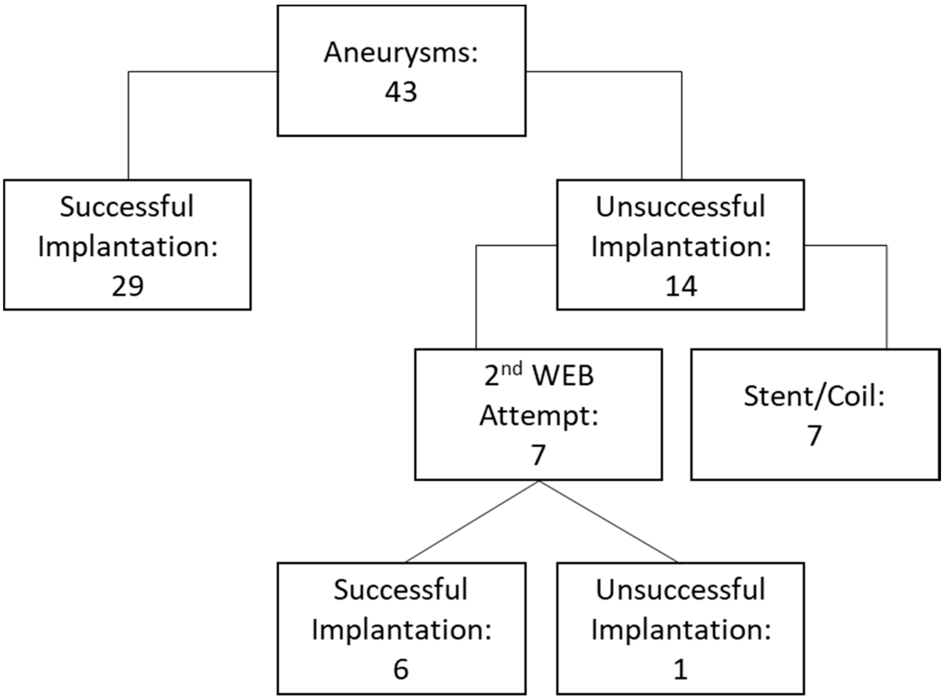

Figure S1. Study flow diagram.

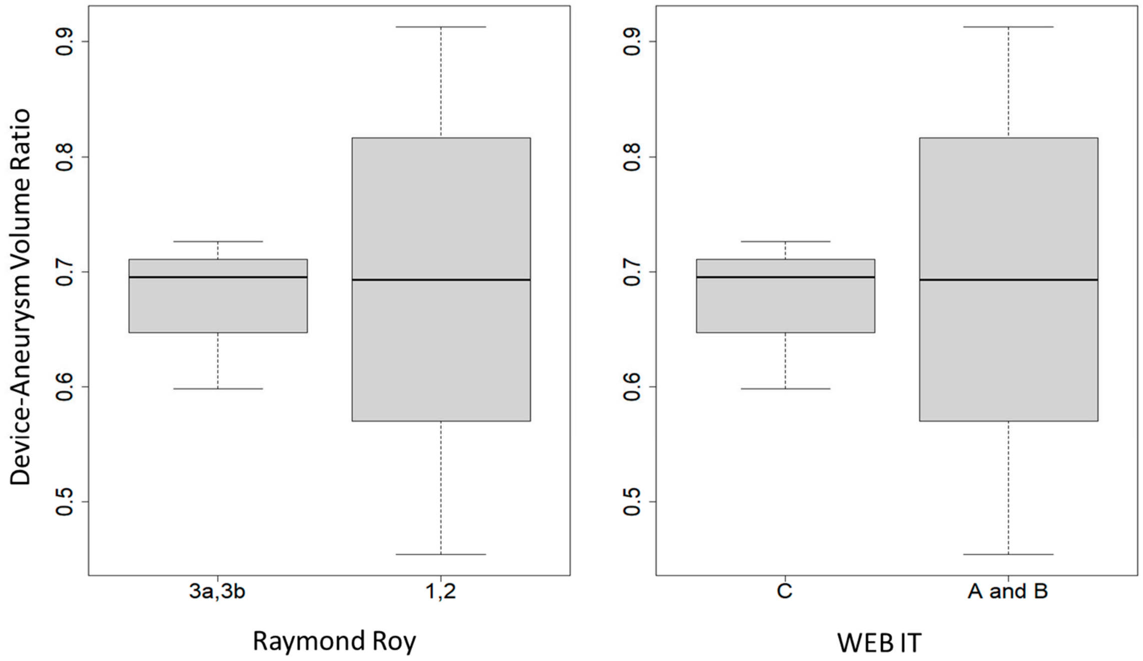

Figure S2. Box and whisker plots showing device-aneurysm volume (DAV) ratios vs obliteration rates at follow-up.

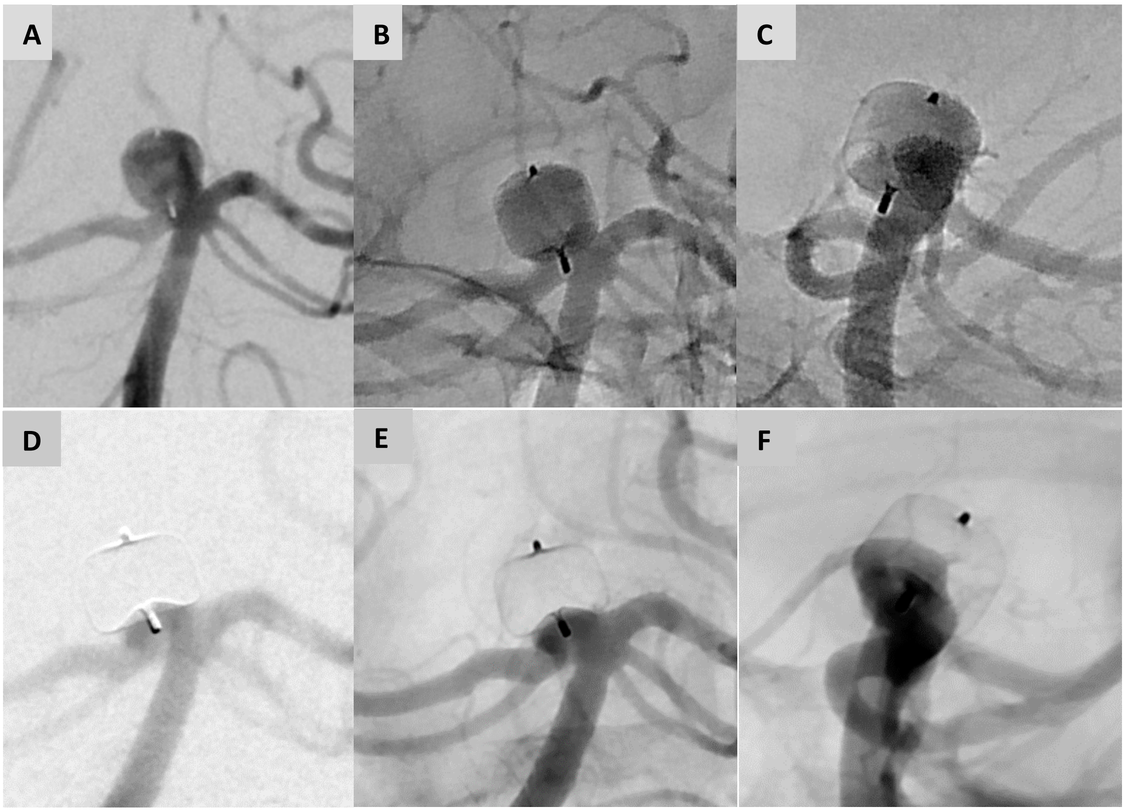

Figure S3. Web compaction. (**A**–**C**) Lateral and anteroposterior (AP) views on intervention day. (**D**–**F**) Lateral and AP views on follow-up.
